# Supplementary material for: Complexome profiling of the Chlamydomonas psb28 mutant reveals TEF5 as an early PSII assembly factor
Source: Plant Cell. 2025 Mar 17;37(6):koaf055. doi: 10.1093/plcell/koaf055 (PMC12164590; doi:10.1093/plcell/koaf055)
Supplement: koaf055_Supplementary_Data [file koaf055_supplementary_data.zip › psb28_interactive.zip!/plots/intensity/qualityPlots/peptides/psaC_Cre-1.g2717046.t1.p1.html]

### Cre-1.g2717046.t1.p1

Trivial name: psaC  
SeqCoverage [median]: 29.60  
Mass: 8.86 kDa  
  
 

| Sequence | MissedCleavage | Mass [Da] | wtIntensity | muIntensity | Score |
| --- | --- | --- | --- | --- | --- |
| ASQMASAPR | 0 | 917.44 | 1107229.95 | 619272.98 | 218.55 |
| SMGLSY | 0 | 656.28 | 264823.79 | 284353.90 | 110.17 |
| VYLGSESTR | 0 | 1010.50 | 2108388.53 | 1531460.87 | 247.10 |
| VYLGSESTRSMGLSY | 1 | 1648.78 | 0.00 | 806.72 | 67.39 |
|  |  |  | 3480442.3 | 2435894.5 |  |
